# Precautionary Measures Against COVID-19 in Residential and Community-Based Facilities: Singapore’s Response

**DOI:** 10.1093/geroni/igaa057.3492

**Published:** 2020-12-16

**Authors:** Vanessa Yong

**Affiliations:** Ministry of Health, Singapore, Singapore

## Abstract

The COVID-19 pandemic has disproportionately infected older adults who are also at higher risks of developing severe health complications and having case fatality if infected. In particular, older adults in residential and community-based facilities are at greater risks of contracting COVID-19, in part due to close proximities and frequent human interactions in these facilities. In Singapore, the Ministry of Health and its implementation agency, the Agency for Integrated Care (AIC), together with long-term care service providers, have jointly developed a number of precautionary measures against COVID-19. This paper defines and describes Singapore’s response in terms of the ‘ABC’ of COVID-19 safety measures for facilities, namely, safe Access, Behaviors, Compounds (i.e. spatial surroundings). These included infection prevention and control measures, access to PPE, distancing and zoning measures, suspension of visitors, alternative accommodation for long-term care workers, and testing to monitor people with long-term care needs and care workers. Incident response teams to support facilities providers in responding to COVID-19 infections were also swiftly set up. The outreach arm of AIC, the Silver Generation Office, further provided information and services support to older people during the pandemic. We share these measures as a set of practices and learning points for other countries undergoing the current pandemic and for future references.

